# Structural brain abnormalities in endothelial nitric oxide synthase‐deficient mice revealed by high‐resolution magnetic resonance imaging

**DOI:** 10.1002/brb3.2801

**Published:** 2022-10-19

**Authors:** Hannah George, Grace V. Mercer, Darcie Stapleton, Laura Dawson, Phillip E. MacCallum, Shoshana Spring, John G. Sled, Jacqueline Blundell, Lindsay S. Cahill

**Affiliations:** ^1^ Department of Chemistry Memorial University of Newfoundland St. John's Canada; ^2^ Department of Psychology Memorial University of Newfoundland St. John's Canada; ^3^ Mouse Imaging Centre Hospital for Sick Children Toronto Canada; ^4^ Translational Medicine Hospital for Sick Children Toronto Canada; ^5^ Department of Medical Biophysics University of Toronto Toronto Canada; ^6^ Discipline of Radiology Memorial University of Newfoundland St. John's Canada

**Keywords:** endothelial nitric oxide synthase, magnetic resonance imaging, mouse, sex differences

## Abstract

**Introduction:**

Endothelial nitric oxide synthase (eNOS) produces nitric oxide, which is essential for a variety of physiological functions in the brain. Previous work has demonstrated the detrimental effects of eNOS deficiency on brain function in male eNOS knockout (eNOS KO) mice. However, the effect of eNOS deficiency on brain structure and any association between these effects and sex is unknown.

**Methods:**

This study used three‐dimensional high‐resolution ex vivo magnetic resonance imaging and behavioral tests of anxiety and cognitive performance to investigate structure–function relationships in the brain of female and male eNOS KO mice in young adulthood.

**Results:**

While there were no differences in anxiety‐like behavior or locomotion, there was a sex‐specific deficit in contextual fear memory retention in male, but not in female, eNOS mice compared to wild‐type controls. Moreover, we found that eNOS deficiency induced changes in multiple brain regions that are involved in learning and fear memory including the hippocampus, amygdala, hypothalamus, and areas of the cortex. Several of these MRI‐detectable neuroanatomical changes were dependent on sex.

**Conclusion:**

The observation that eNOS deficiency impacts brain structure at an early age demonstrates the importance of eNOS for healthy brain development.

## Graphical abstract

Using behavioural tests of anxiety and cognitive function and high‐resolution ex vivo magnetic resonance imaging, we demonstrated the endothelial nitric oxide synthase deficiency in mice impacts brain structure and function in a sex dependent manner.

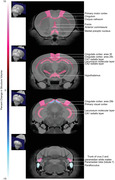

## 1 INTRODUCTION

The importance of nitric oxide (NO) for physiological functions such as neural development, vasodilation, and vascular remodeling is well established (Guix et al., 2005; Rudic et al., 1998). NO is predominantly produced from l‐arginine by the enzyme nitric oxide synthase (NOS). In the brain, endothelial NOS (eNOS) is expressed in endothelial and neuronal cells in areas such as the olfactory bulb, cortex, amygdala, and cerebellum, and expressed in pyramidal cells in the CA1 area of the hippocampus (Caviedes et al., 2017; Hara et al., 1996; Roskams et al., 1994; Son et al., 1996). Several studies have used an eNOS knockout (eNOS KO) mouse to study the effects of eNOS deficiency on brain function. For example, eNOS KO mice showed deficits in long‐term potentiation (LTP) in the hippocampus (Wilson et al., 1998, 1997), cerebral cortex (Haul et al., 1999), and the cortico‐striatal circuit (Doreulee et al., 2003). In addition, eNOS KO mice displayed abnormal vascular tone in the carotid arteries (Faraci et al., 1998; Lamping & Faraci, 2001). A recent review presents partial eNOS deficient mice as a model of spontaneous cerebral small‐vessel disease, with evidence of cerebral hypoperfusion and blood‐brain barrier leakage early in life (Liao et al., 2021). Considering these functional impairments in the eNOS deficient brain, we hypothesized that there would be neuroanatomical differences in eNOS KO mice and that these structural abnormalities would correlate with behavioral outcomes. Three‐dimensional high‐resolution magnetic resonance imaging (MRI), in combination with deformation‐based morphometry, can be used to investigate the macroscopic changes in brain structure that occur in eNOS deficient mice and provide new information about the role of eNOS expression in neuroanatomy.

Previous studies have implicated eNOS in learning and memory, aggression, anxiety, and locomotion (Austin et al., 2013; Demas et al., 1999; Dere et al., 2002; Frisch et al., 2000; Liao et al., 2021). These studies have used only male mice to study the eNOS phenotype. The influence of eNOS deficiency on behavior in female mice is unknown. Cerebrovascular tone was more affected in female than male eNOS KO mice (Lamping & Faraci, 2001), indicating the impact of eNOS deficiency on brain structure and behavior may be dependent on sex.

The present study aims to understand further the structure–function relationships of the adult brain in eNOS deficient mice using three‐dimensional high‐resolution ex vivo MRI and behavioral testing of anxiety, exploratory behavior, and learning and memory in both females and males. MRI and deformation‐based morphometry provide unbiased, whole‐brain analysis, allowing us to perform a comprehensive assessment of the effect of eNOS deficiency on neuroanatomy.

## 2 METHODS

### 2.1 Animals

C57BL/6J mice (stock No.000664) as wild‐type (WT) controls and eNOS KO (stock No.002684) were obtained from Jackson Laboratories. All mice were given ad libitum access to food and water in standard laboratory conditions on a 12‐h light‐dark cycle. To assess whether eNOS deficiency had an impact on neurodevelopment, 20 C57BL/6J (10 females and 10 males) and 20 eNOS KO (10 females and 10 males) adult mice (8–9 weeks of age) underwent a battery of behavioral tests including the elevated plus maze and open field to test anxiety‐like behavior and exploratory behavior, social interaction to test social anxiety, and fear conditioning to test learning and memory. To detect changes in neuroanatomy associated with eNOS deficiency and whether behavioral outcomes related to regional brain volume, the same cohort of mice underwent three‐dimensional high‐resolution ex vivo MRI. All animal experiments were approved by the Institutional Care Committee at Memorial University of Newfoundland and conducted in accordance with guidelines established by the Canadian Council on Animal Care.

### 2.2 Behavioral assays

Behavioral tests were conducted in dedicated behavioral testing rooms during the standard light phase. Prior to all tests, mice were habituated to the testing room for at least 30 min. Mice were tested over 5 days in the following sequence: elevated plus maze, open field, social interaction, and fear conditioning.

#### 2.2.1 Elevated plus maze

The elevated plus maze apparatus was made of 0.6 cm thick white plexiglass and consisted of four arms arranged at right angles in the shape of a plus sign (Adamec et al., 2006, 2004). Each arm was 29.0 cm long and 5.1 cm wide, and there was a 10.2 cm^2^ square in the center. The two closed arms opposite each other had 14 cm high walls enclosing them. The two open arms had very small 0.5 cm high walls around all sides. Mice were placed in an open arm facing away from the center platform and allowed a 5 min period to explore. The EthoVision XT10 tracking system (Noldus, Wageningen, the Netherlands) was used to track the total distance the mice traveled, the mean velocity, the time the mice spent in the open and closed arms, and the number of entries into the open and closed arms. Ratio time was calculated as the time spent in the open arms of the maze divided by the time spent in both the open and closed arms of the maze. Ratio frequency was calculated as the number of entries into the open arms of the maze divided by the number of entries in all arms of the maze.

#### 2.2.2 Open field activity

The open field apparatus was a 48 × 48 × 48 cm gray box. To determine the amount of time the mice spent in the center of the box relative to the perimeter, a piece of electrical tape marked a box on the floor 10 cm from the walls of the arena. Mice were placed in the center of the box and allowed 5 min to explore. EthoVision XT10 was used to track the mice and measure the amount of time the mice spent in the center, the total distance traveled, and the mean velocity.

#### 2.2.3 Social interaction

The social interaction test (Golden et al., 2011) was completed in the open field arena. The test consisted of two trials, either with or without a novel C57BL/6J mouse present. In the first trial, an empty metal cage was positioned against one of the walls, and mice were placed in the center of the arena and allowed to explore for 150 s. After this period, the test mice were removed from the box, and a novel male trial mouse was placed inside the metal cage within the arena. Test mice were then promptly returned to explore the arena for a second trial of 150 s in the presence of the social target. The arena is divided into two areas: an interaction zone surrounding the target mouse and the rest of the box. EthoVision XT10 was used to track and measure the amount of time and frequency in the interaction zone during both trials. The social interaction ratio was calculated by dividing the time spent in the interaction zone when the novel trial mouse was present by the time spent in the interaction zone when the target was absent.

#### 2.2.4 Fear conditioning

The fear conditioning apparatus was a plexiglass shock box with clear front and rear walls (Coulbourn Instruments, Holliston, MA, USA) (Curzon et al., 2009; MacCallum & Blundell, 2020). For training, mice were placed in the box for a 5–6 min period which consisted of a 1.5‐2 min acclimation period followed by a 30‐s 80 dB noise that terminates with a 2–3 s 0.7 mA foot shock. There was a 1 min interstimulus interval, and the shock‐tone pairing was repeated. The mice remained in the chamber for 1.5‐2 min after the last termination of the last tone‐shock pairing. Twenty‐four hours later, mice were re‐exposed to the same chamber for 4 min to test freezing in response to the chamber (i.e., contextual fear memory). No shocks or tones were delivered. The next day, cued auditory recall was tested by exposing the mice to a modified chamber (vanilla extract blotted over surfaces, wooden floor over the steel bar shock floor, and cardboard walls) for 2 min with no tone and no shocks, followed by the same 80 dB noise used in conditioning for 2 min. Videos were manually scored for freezing behavior by one reviewer, blinded to the genotype of the animals. Four mice had to be excluded because of instrumentation failure during testing (three male WTs and one female WT).

### 2.3 Brain sample preparation

Upon completion of behavioral tests, adult mice (10–11 weeks old) were prepared for ex vivo MRI by transcardiac perfusion (Cahill et al., 2012; Spring et al., 2007). Mice were anesthetized via intraperitoneal ketamine/xylazine injections (150 and 10 mg/kg, respectively). Thoracic cavities were opened and through the left ventricle, mice were perfused with 30 ml of phosphate‐buffered saline (PBS), 1 μl/ml heparin, and 2 mM of a gadolinium MRI contrast agent (ProHance, Bracco Diagnostics Inc.) at a rate of 1 ml/min. This was followed by infusion with 4% paraformaldehyde (PFA) and 2 mM ProHance in PBS. Following perfusion, the heads were removed, and the brains within the skull were postfixed in 4% PFA with 2 mM ProHance overnight at 4°C. Brain samples were stored in a solution of PBS, 2 mM ProHance, and sodium azide.

### 2.4 Ex vivo magnetic resonance imaging

Images were acquired using a multi‐channel 7.0 T magnet (Varian Inc. Palo Alto, CA, USA) located at the Mouse Imaging Centre at the Hospital for Sick Children (Toronto, ON, USA). Sixteen samples were imaged simultaneously using a custom‐built solenoid array (Dazai et al., 2011). Anatomical scans were performed using the following optimized parameters: a T2‐weighted, three‐dimensional fast spin‐echo sequence using a cylindrical *k‐*space acquisition (Nieman et al., 2005) with TR = 350 ms, TE = 12 ms, echo train length = 6, four averages, field‐of‐view = 20mm × 20mm × 25mm, matrix size = 504 × 504 × 630, isotropic image resolution = 40 μm (Spencer Noakes et al., 2017).

An automated image registration approach (Nieman et al., 2018) using the Pydpiper toolkit (Friedel et al., 2014) and the advanced normalization tools deformation algorithm (Avants et al., 2009) was used to assess anatomical differences related to eNOS deficiency. The 40 MR images were registered using a series of linear (six parameters, then 12 parameters) and nonlinear registration steps to create an average image (Nieman et al., 2018). The registration yielded deformation fields for each individual brain and the Jacobian determinants provided an estimate of the local volume changes at every voxel in the brain. Using the results of the linear alignment, multiple templates of a segmented anatomical atlas (Dorr et al., 2008; Qiu et al., 2018; Richards et al., 2011; Steadman et al., 2014; Ullmann et al., 2013) with 182 labelled brain structures were created (the MAGeT procedure; Chakravarty et al., 2013). Multiple templates were used to improve the segmentation accuracy for the structure volumes. From the final voted segmentation, absolute volumes were calculated for each image.

### 2.5 Statistical analysis

All statistical tests were performed using RMINC (https://github.com/Mouse‐Imaging‐Centre/RMINC) and R statistical software (www.r‐project.org). The behavioral outcomes were analyzed using a two‐way analysis of variance (ANOVA) with main effects of genotype and sex and allowing for interaction between the two. If the ANOVA was significant, Tukey's post hoc tests were performed. A value of *p* < .05 was taken to be significant. To detect neuroanatomical differences from the ex vivo MRI images, we performed two types of image analysis: voxel‐wise and volumetric (structure‐wise) analysis. These two approaches can provide different but complementary information about focal and region‐specific changes in morphometry (Lerch et al., 2017). A linear model was used at every voxel and for each structure with the main effects of genotype, sex and genotype‐by‐sex interaction. Multiple comparisons were controlled for using the false discovery rate (FDR) (Genovese et al., 2002), and statistical significance was defined at an FDR threshold of 10%. To identify potential relationships between behavior and absolute brain structure volumes, an exploratory analysis was performed. For the correlation analysis, the behavioral outcomes were limited to the fear conditioning tests as this was the only assay that showed differences between genotypes and the MRI‐based structure volumes were limited to regions that were significantly different between WT and eNOS KO mice at an FDR threshold of 10%. The behavioral outcomes of the fear conditioning test were analyzed using a two‐way ANOVA with main effects of brain structure volume and genotype. A value of *p* < .05 was taken to be significant. If the ANOVA was significant, a Pearson's correlation test was used to assess the extent to which the structure volume could explain the behavioral outcome. As these correlations were exploratory, no corrections for multiple comparisons were made.

## 3 RESULTS

### 3.1 Behavioral results

**FIGURE 1:**
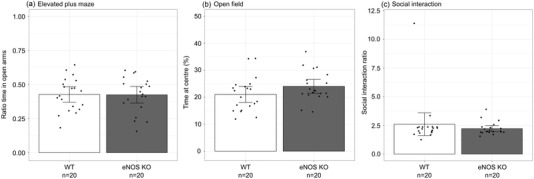
There were no significant differences between endothelial nitric oxide synthase knockout (eNOS KO) (gray bars) and wild‐type (WT) mice (white bars) observed in the (a) ratio of time spent in the open arms of the elevated plus maze, (b) percentage of time spent in the center of the open field, and (c) social interaction test. Data are shown as means and 95% confidence intervals. *n* refers to the number of mice (10 females and 10 males per group).

Anxiety‐like behavior and locomotion was assessed using an elevated plus maze and an open field test. In the elevated plus maze, there was no effect of genotype or sex on the ratio time spent in the open arms (*p*_genotype_ = .9, *p*_sex_ = .7, Figure 1a), the ratio frequency of entries in the open arms (*p*_genotype_ = .9, *p*_sex_ = .5, Figure S1a), or in the number of total entries (*p*_genotype_ = .9, *p*_sex_ = .4, Figure S1b). There was no effect of genotype or sex on locomotion or activity level, measured as the total distance travelled and the mean velocity in the elevated plus maze (distance travelled: *p*_genotype_ = .9, *p*_sex_ = .5; mean velocity: *p*_genotype_ = .8, *p*_sex_ = .8, Figure S1c,d) and the open field (distance travelled: *p*_genotype_ = .9, *p*_sex_ = .9; mean velocity: *p*_genotype_ = .9, *p*_sex_ = .9, Figure S1e,f). In the open field test, the percentage of total time spent in the center was not dependent on genotype or sex (*p*_genotype_ = .1, *p*_sex_ = .9, Figure 1b). These results are consistent with the social interaction test to assess social anxiety where there was no effect of genotype or sex on preference to spend time interacting with a social target (*p*_genotype_ = .4, *p*_sex_ = .5, Figure 1c).

**FIGURE 2:**
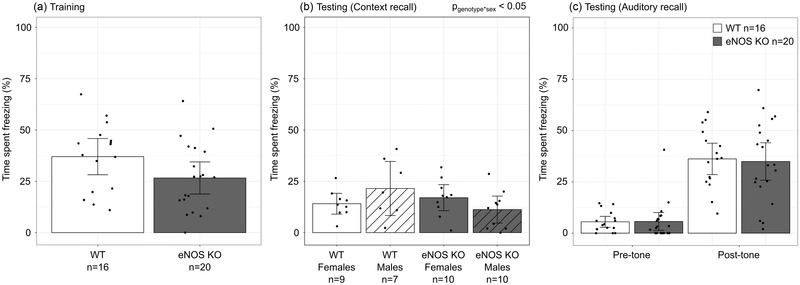
(a) There was no significant difference in freezing between endothelial nitric oxide synthase knockout (eNOS KO) (gray bar) and wild‐type (WT) mice (white bar) trained on the cued fear‐conditioning paradigm. (b) After training, there was a significant sex‐by‐genotype interaction (*p*_genotype*sex_ < .05) following exposure to the training context, with no difference in freezing between female eNO KO (gray bar) and female WT mice (white bar) and a 48% decrease in freezing in male eNOS KO (gray hatched bar) compared to male WT mice (white hatched bar) (*p* = .08). (c) There was no significant difference in freezing between eNOS KO (gray bars) and WT mice (white bars) before or after re‐exposure to the conditioning tone cue. Data are shown as means and 95% confidence intervals. *n* refers to the number of mice.

During the fear conditioning training, there were no significant effects of genotype or sex on the freezing behavior prior to the first shock‐tone pairing (eNOS KO: 0.8% [CI: 0.1–1.5] vs. WT: 0.5% [CI: 0.0–1.0]). While there was no sex effect, there was a trend toward a decrease in the fear response immediately after the last shock‐tone pairing in the eNOS KO mice compared to the WTs (*p* = .07, Figure 2a). Contextual and cue fear memory was assessed 2 or 3 days post‐training. There was no effect of genotype or sex on context memory retention; however, there was a significant sex‐by‐genotype interaction (*p* < .05, Figure 2b). Post hoc analysis using a *t*‐test showed that the context memory retention was not different between the genotypes for females. Although memory retention was 48% lower in male eNOS KO mice compared to WT mice, this did not reach statistical significance (*p* = .08). While both genotypes showed statistically significant memory from the conditioning tone cue playback, there was no effect of genotype or sex on cued fear memory consolidation (*p*_genotype_ = .8, *p*_sex_ = .2, Figure 2c).

### 3.2 MRI results

**FIGURE 3:**
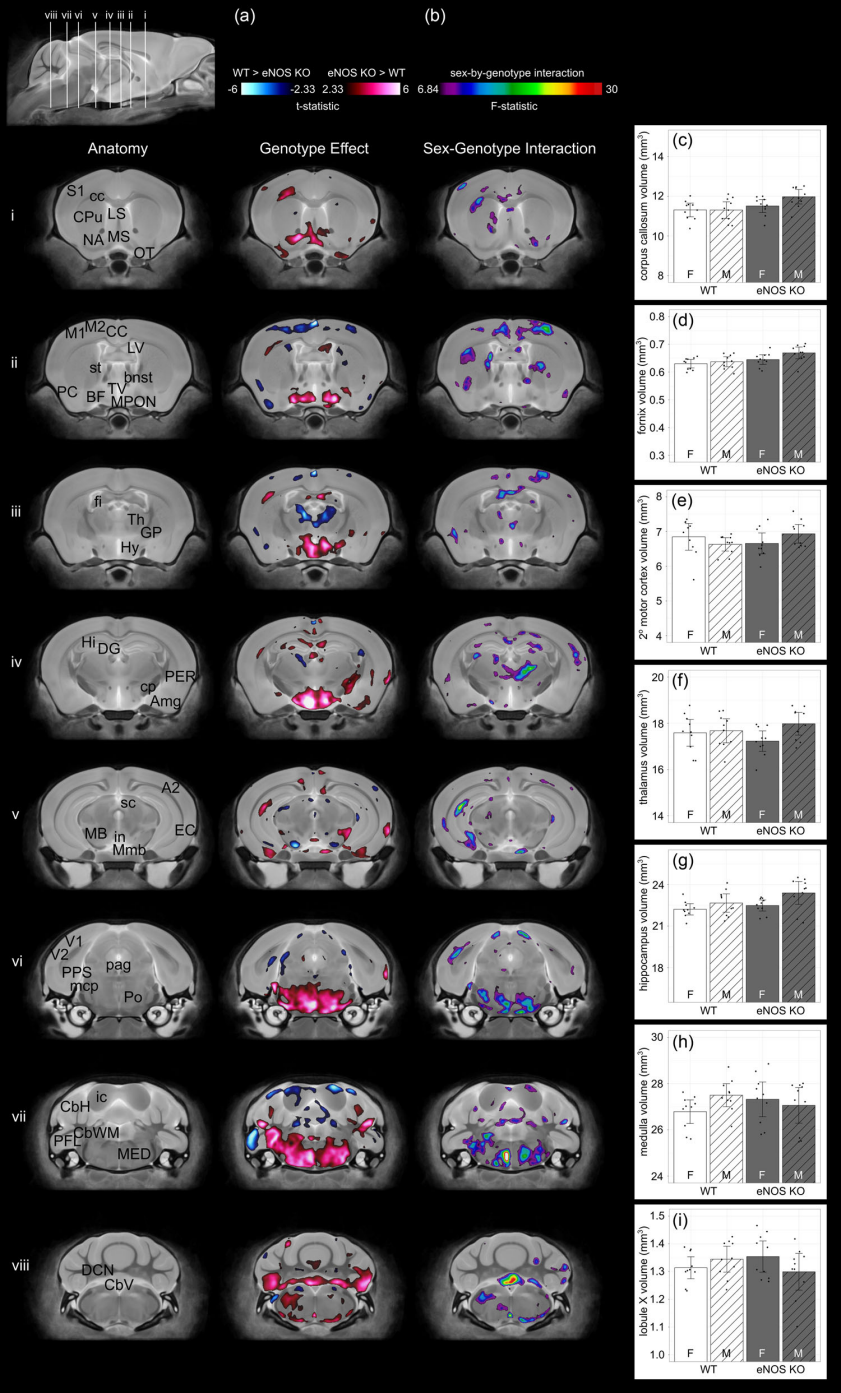
(a) Representative coronal slices comparing anatomical differences in the brains of wild‐type (WT) and endothelial nitric oxide synthase knockout (eNOS KO) mice. The images are overlaid with color maps indicating regions of absolute volume that are significantly larger (hot colors) and significantly smaller (cool colors) in eNOS mice compared to WTs (10% false discovery rate [FDR]). (b) Representative coronal slices showing a significant sex‐by‐genotype interaction (*F*‐statistic = 6.84, 10% FDR). (c–i) Absolute volumes of several brain structures (from segmented atlas‐based analysis) that reflect the sex‐by‐genotype interaction. Data shown as means and 95% confidence intervals. *n* = 10 mice per sex per group. Abbreviations: A2, secondary auditory cortex; Amg, amygdala; BF, basal forebrain; bnst, bed nucleus of stria terminalis; CbH, cerebellar hemisphere; CbV, cerebellar vermis; CbWM, cerebellar white matter; CC, cingulate cortex; cc, corpus callosum; cp, cerebral peduncle; CPu, caudate/putamen; DCN, deep cerebellar nuclei; DG, dentate gyrus; EC, entorhinal cortex; fi, fimbria; GP, globus pallidus; Hi, hippocampus; Hy, hypothalamus; ic, inferior colliculus; in, interpeduncular nucleus; LV, lateral ventricle; LS, lateral septum; M1, primary motor cortex; M2, secondary motor cortex; MB, midbrain; mcp, middle cerebellar peduncle; MED, medulla; Mmb, mammillary bodies; MPON, medial preoptic nucleus; MS, medial septum; NA, nucleus accumbens; OT, olfactory tubercle; pag, periaqueductal gray; PC, piriform cortex; PER, perirhinal cortex; PFL, paraflocculus; Po, pons; PPS, pre‐parasubiculum; S1, primary somatosensory cortex; sc, superior colliculus; st, stria terminalis; Th, thalamus; TV, third ventricle; V1, primary visual cortex; V2, secondary visual cortex

Analysis of brain morphology using ex vivo MRI revealed significant differences in specific brain regions of the adult eNOS KO mice compared to WTs (*n* = 10/group/sex). While there was no difference in whole brain volume between genotype (eNOS KO: 451 mm^3^ [CI: 444–458] vs. control: 445 mm^3^ [CI: 440–450], *p* = .2), a voxelwise comparison between eNOS KO and control mice demonstrated significant absolute volume differences in regions in the gray and white matter of the brain (Figure 3a). Areas of the brain that demonstrated a decrease in volume in eNOS KO mice were areas of the cortex (primary and secondary motor cortex, piriform cortex, cingulate cortex, secondary auditory cortex), thalamus, midbrain, pre‐parasubiculum, inferior and superior colliculus, and paraflocculus. Other areas of the brain were larger in eNOS KO mice, including regions in the cortex (primary somatosensory cortex, entorhinal cortex, perirhinal cortex), nucleus accumbens, caudate/putamen, basal forebrain, corpus callosum, olfactory tubercle, medial preoptic nucleus, lateral ventricle, third ventricle, hypothalamus, globus pallidus, fimbria, dentate gyrus, hippocampus (CA1 radialis layer, CA1 lacunosum molecular layer, CA1 pyramidal layer, CA2 radialis layer, CA3 radialis layer, CA oriens layer), amygdala, cerebral peduncle, mammillary bodies, interpeduncular nucleus, periaqueductal gray, pons, medulla, middle cerebral peduncle, and cerebellum (areas in the cerebellar hemisphere [e.g., crus I and II of the ansiform lobule, paramedian lobule, simple lobule, copula of pyramis], areas in the cerebellar vermis [e.g., lobule III, lobule IV‐V, lobule VI, lobule VII, lobule VIII, lobule X] and in the cerebellar vermis white matter). A significant sex‐by‐genotype interaction demonstrated that several of the differences between genotypes were dependent on sex (*F*‐statistic = 6.84, 10% FDR, Figure 3b). For example, the corpus callosum, fornix, and hippocampus were larger in only male eNOS KO mice (Figure 3c,d,g). The thalamus was smaller in only female eNOS KO mice (Figure 3f). While the volume of several brain regions in the WTs showed expected sexual dimorphisms, the volumes for the eNOS KO mice were reversed. Specifically, the medulla and the cerebellum are known to be larger in males than females (Qiu et al., 2018; Spring et al., 2007); however, in the eNOS mice the medulla and lobule X of the cerebellum were larger in females than males (Figure 3h,i). While not established as a sexually dimorphic region, this reversed female–male difference in the eNOS KO mice was also true for the secondary motor cortex (Figure 3e).

**FIGURE 4:**
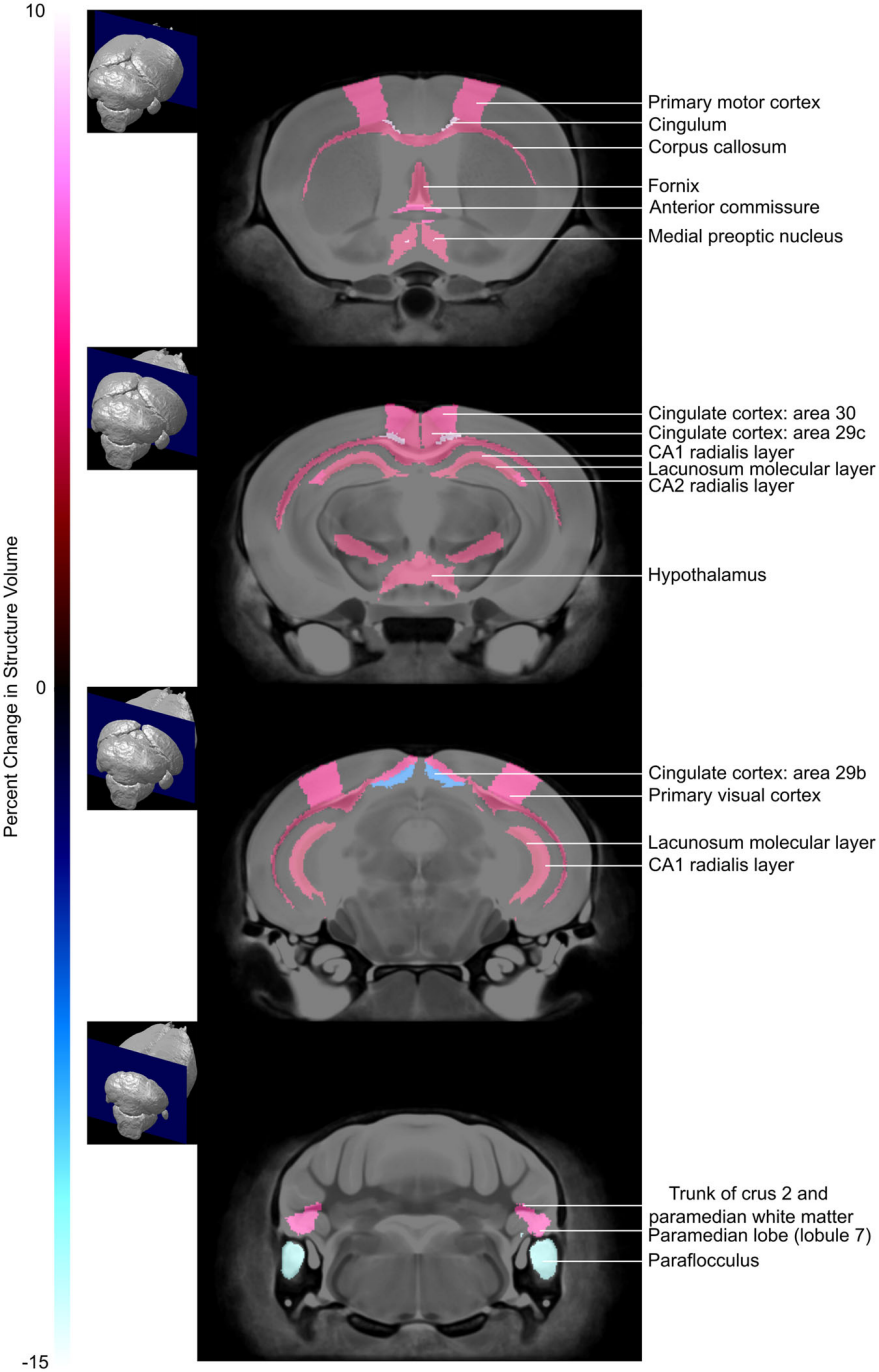
Coronal magnetic resonance imaging (MRI) slices showing the differences in absolute structure volume between endothelial nitric oxide synthase knockout (eNOS KO) and control mice (false discovery rate [FDR] 10%). Slices arranged from the anterior (top) to posterior (bottom). The percentage change in brain structure volume is indicated by the color scale bar on the left.

We also evaluated brain morphology using a segmented atlas. There was no significant sex‐by‐genotype interaction for the absolute brain structure volumes and therefore males and females were not evaluated separately. Of the 182 segmented brain structures, 14% (26/182) were found to be significantly different in absolute volume (mm^3^) between genotypes at an FDR of 10% (Figure 4 and Table 1).

**TABLE 1:** Absolute volume of brain structures that were significantly different in endothelial nitric oxide synthase knockout (eNOS KO) compared to wild‐type (WT) mice

Structure	Change in absolute volume (%)	Groupwise absolute volume difference: Uncorrected *p*‐value
Anterior commissure	5.4	.0003**
Cerebellum: Paramedian lobule (lobule 7) Paramedian lobule (white matter) Truck of crus 2 and paramedian white matter Paraflocculus Paraflocculus white matter Flocculus white matter		
	5.7	.01*
	9.0	.001**
	5.2	.006*
	−12.4	.0002***
	−7.5	.002**
	8.2	.01*
Corpus callosum	3.9	.01*
Cortex: Cingulate cortex: Area 24a' Cingulate cortex: Area 29b Cingulate cortex: Area 29c Cingulate cortex: Area 30 Dorsolateral orbital cortex Frontal association cortex Lateral orbital cortex Primary motor cortex Primary visual cortex		
	8.4	.005*
	−7.2	.009*
	4.1	.01*
	4.7	.008*
	6.3	.005*
	4.3	.00005***
	5.1	.0001***
	4.5	.002**
	4.9	.01*
Cingulum	9.1	.00003***
Fornix	3.7	.008*
Hippocampus: Lacunosum molecular layer CA1 radialis layer CA2 radialis layer		
	3.4	.007*
	3.7	.01*
	4.6	.008*
Hypothalamus	3.8	.003**
Lateral olfactory tract	4.0	.0003**
Medial preoptic nucleus	9.1	.0007**
Subependymale zone/rhinocele	6.4	.005*

*Note*: *n* = 20 mice per group (there was no significant sex‐by‐genotype interaction and therefore males and females were not evaluated separately).

*FDR = 10%; **FDR = 5%; ***FDR = 1%.

### 3.3 Behavior correlations with brain volume

**FIGURE 5:**
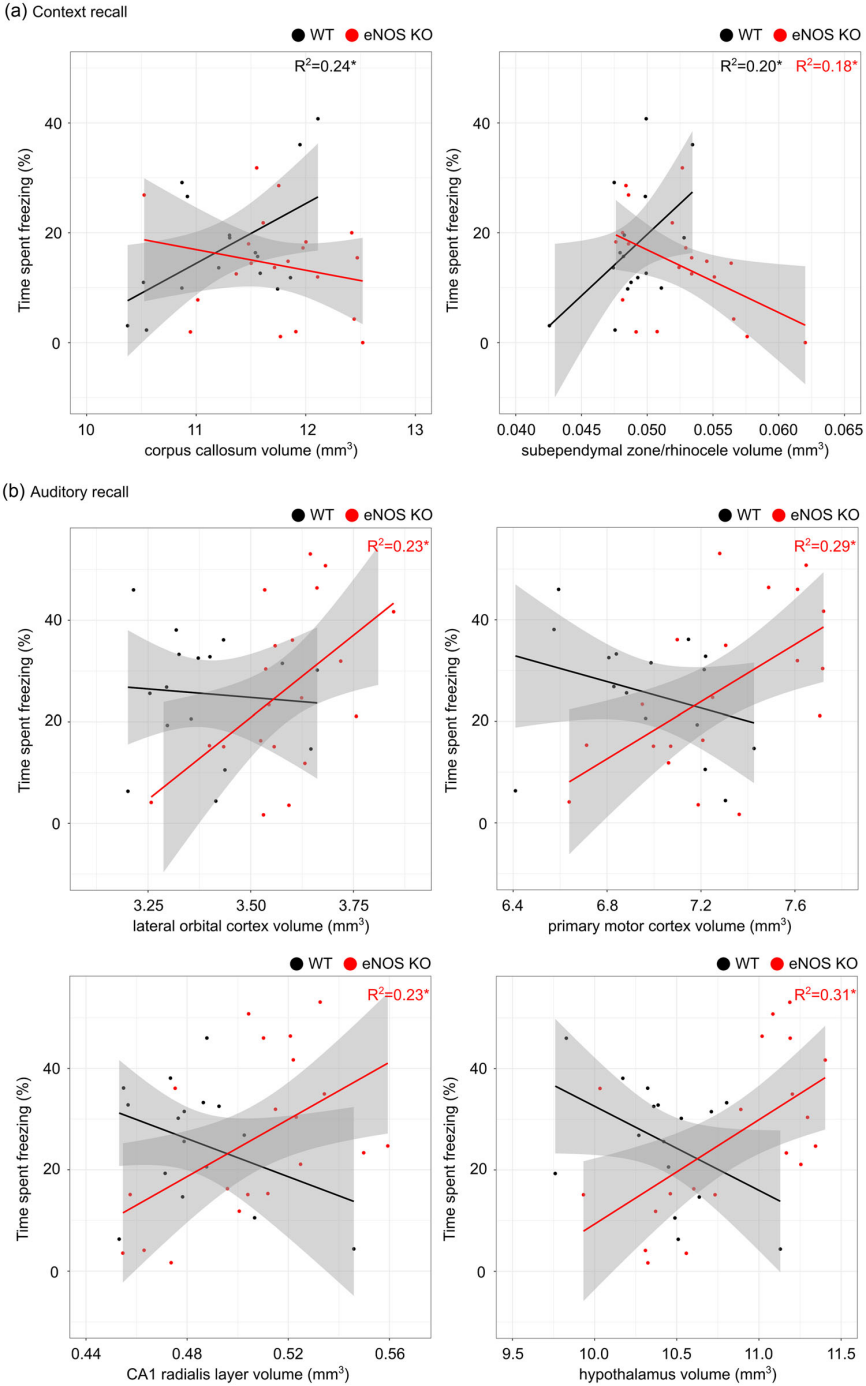
Correlation analysis between brain regions and behavior outcomes from fear conditioning in endothelial nitric oxide synthase knockout (eNOS KO) (red, *n* = 20) and wild‐type (WT) (black, *n* = 16) mice. There was no significant sex‐by‐genotype interaction for the absolute brain structure volumes and therefore males and females were not evaluated separately. Scatterplots showing the correlation between time spent freezing during (a) context recall testing and the volume of the corpus callosum and subependymal zone/rhinocele and (b) auditory recall testing and the volume of the lateral orbital cortex, primary motor cortex, CA1 radialis layer and hypothalamus. The shaded gray area represents the 95% confidence interval. The correlation was assessed by the Pearson's *R* test (adjusted‐*R* ^2^). *p < .05

To investigate the relationship between the neuroanatomical differences and the behavioral findings, we performed an exploratory analysis between the fear conditioning outcomes and the brain regions that were determined to be statistically different between WT and eNOS KO mice (Table 1). For context memory recall, while the WT mice showed a positive correlation between freezing and the volume of the corpus callosum and the subependymal zone/rhinocele, the eNOS KO mice showed no correlation with the corpus callosum volume and a significantly negative correlation with the subependymal zone/rhinocele (Figure 5a). On the other hand, for auditory cued memory recall, there were no associations between structure volumes and freezing in the WT mice; however, the eNOS KO mice showed a positive correlation between freezing and the volume of several structures including the lateral orbital cortex, primary motor cortex, CA1 radialis layer, and the hypothalamus (Figure 5b).

## 4 DISCUSSION

Using behavioral testing and high‐resolution ex vivo MRI, we examined the effect of eNOS deficiency on neurological function and macroscopic brain structure. The eNOS KO mice did not show any differences when compared to WT controls in social or environmental anxiety, in exploratory behavior, or locomotion. There was a trend toward a decrease in the fear response immediately after the shock‐tone pairings in the eNOS KO mice, suggesting a possible impairment in reaction to aversive shock. This trend is likely not a result of a decrease in pain sensitivity, with eNOS KO mice displaying the same pain behavior as WT controls (Boettger et al., 2007). Despite this trend, the eNOS KO mice showed the expected memory recall following the tone cue. However, the male, and not the female, eNOS KO mice showed a decrease in context memory retention. In contrast to our results, Frisch et al. (2000) found the eNOS KO mice were less active in the open field and showed increased anxiety in the elevated plus maze. The smaller sample size (*n* = 14 compared to *n* = 40 presently) and age of the mice (12 weeks compared to 8–9 weeks presently) may explain the differences in behavioral outcomes. In older mice (18–22 months), Dere et al. (2002) reported reduced exploration in the open field and while there was no anxiety phenotype in the eNOS KO mice, they did show a decrease in locomotion compared to WT controls. The findings with respect to eNOS deficiency on learning and memory are heterogenous with improved learning reported for eNOS KO mice in the Morris water maze (Frisch et al., 2000), impaired learning in a radial arm maze (Austin et al., 2013), and no differences in learning the radial arm maze (Dere et al., 2001) or the Morris water maze (Hendrickx et al., 2021; Liao et al., 2021). Our results showed a trend toward impairments in context recall in only male eNOS KO mice. To our knowledge, this is the first report of fear conditioning results in eNOS deficient mice.

A striking feature of the present study is the number of brain regions affected in early adulthood by eNOS deficiency. A study using autoradiography and tissue sections in WT male C57BL/6J mice has shown that eNOS is distributed in the olfactory bulb, tenia tecta, rhinal fissure, amygdala, cerebellum, hippocampus, dentate gyrus, cortex, and superior colliculus (Hara et al., 1996). Many of these brain regions were found, by either voxelwise or structure volume analysis, to be significantly different in eNOS KO mice compared to WTs. In addition, the volume of several brain regions that were not found to have high levels of eNOS were affected by eNOS deficiency including the hypothalamus and white matter structures including the cingulum and corpus callosum. NO is essential for hypothalamic function and is primarily supplied by neuronal NOS (nNOS) (Chachlaki et al., 2017). However, our data suggest that a global deficiency in eNOS can impact the structure of the hypothalamus, potentially affecting its function. White matter pathology, indicated by myelin loss and reduced mature oligodendrocytes, has been reported for older eNOS deficient mice (>12 months of age) (Liao et al., 2021).

While there was no overt abnormal behavior observed in the eNOS KO mice, several of the affected brain regions in the eNOS deficient mice are implicated in learning and fear memory. For example, regions of the hippocampus, amygdala, and cortex were found to be associated with fear memory retrieval in a study of neural activation using serial two‐photon tomography (Vousden et al., 2015). For contextual fear memory acquisition and retrieval, the involvement of the hippocampus (Huff et al., 2006; Lee & Kesner, 2004; Mamiya et al., 2009), cingulate cortex (Frankland et al., 2004), and perirhinal cortex (Corodimas & LeDoux, 1995) are well‐established. Auditory fear memories are associated with the hippocampus (Mamiya et al., 2009), amygdala (Ploski et al., 2008), hypothalamus (LeDoux et al., 1988), auditory cortex (LeDoux, 2000), ventrolateral orbitofrontal cortex (Zimmerman et al., 2018), primary motor cortex (Xu et al., 2019), and somatosensory cortex (Wei et al., 2002). Moreover, we found that the structural MRI results for several of these brain regions were differentially associated with the behavioral findings from the fear conditioning test for eNOS KO and WT control mice. For example, the volume of the lateral orbital cortex, primary motor cortex, CA1 radialis layer of the hippocampus, and hypothalamus were positively associated with the freezing behavior in the auditory memory recall test for the eNOS mice, explaining 23%–31% of the variance in this behavior, with no correlation for the WT control mice. We found that the subependymal zone/rhinocele is negatively associated with context recall in the eNOS KO mice. This brain region has not been discussed in relation to fear memory retrieval but is known to be important for neurogenesis, sending neurons to the olfactory bulbs and glial cells to the cortex and corpus callosum (Kazanis, 2009). Given the brain regions that are most impacted by eNOS deficiency, tests of olfaction and motor coordination in eNOS KO mice will be the subject of future investigations.

A strength of this study is the use of both female and male mice in the experimental design. To date, the studies that have investigated behavioral outcomes in eNOS deficient mice have only included males. Our study revealed sex‐specific differences in memory and in brain structure volumes. Deficiency in eNOS preferentially impacted retrieval in contextual fear conditioning and the volume of brain regions such as the corpus callosum, fornix, and hippocampus in male mice. These sex differences are consistent with a study by Li et al. (2004) that looked at both female and male eNOS KO mice in the context of cardiac dysfunction. They reported a higher prevalence of cardiovascular defects and increased mortality in older male eNOS KO mice. The present study adds to the growing literature that demonstrates the importance of examining both sexes in brain and behavior studies.

In summary, the use of a genetic mouse model and three‐dimensional ex vivo high‐resolution anatomical MRI provided an opportunity to examine the relationship between structural changes in the brain and eNOS deficiency. While older mice that are deficient in eNOS have been studied as a model of stroke (Cui et al., 2009; Tan et al., 2015) and Alzheimer's disease‐related pathologies (Austin et al., 2013), the results of the present study show evidence of sex‐specific brain pathology in young adulthood, before the onset of overt behavioral abnormalities. To properly test interventions for disease prevention using eNOS KO mice, experiments would need to begin at an early age. Our data provide new insights into the importance of eNOS for normal brain neuroanatomy. Future studies of disease pathology could benefit from the use of an inducible eNOS KO mouse model to study eNOS deficiency in a tissue‐specific and time‐controlled manner.

## CONFLICT OF INTEREST

The authors declare no conflict of interest.

### ETHICS STATEMENT

All animal experiments were approved by the Institutional Care Committee at Memorial University of Newfoundland and conducted in accordance with guidelines established by the Canadian Council on Animal Care.

### PEER REVIEW

The peer review history for this article is available at: https://publons.com/publon/10.1002/brb3.2801.

## Supporting information

Supplementary Figure 1. There were no significant differences between eNOS KO (gray bars) and WT mice (white bars) observed in the (a) ratio frequency of entries in the open arms of the elevated plus maze, (b) total entries (open or closed) in the elevated plus maze. There were also no significant differences between genotypes in the distance travelled in (c) the elevated plus maze or (e) the open field or in the mean velocity in (d) the elevated plus maze or (f) open field. Data shown as means and 95% confidence intervals. n refers to the number of mice (10 females and 10 males per group)

## Data Availability

The datasets generated during the current study are available from the corresponding author on reasonable request.
