# Supplementary figures and images for: Structural brain abnormalities in endothelial nitric oxide synthase‐deficient mice revealed by high‐resolution magnetic resonance imaging
Source: Brain Behav. 2022 Oct 19;12(11):e2801. doi: 10.1002/brb3.2801 (PMC9660425; doi:10.1002/brb3.2801)

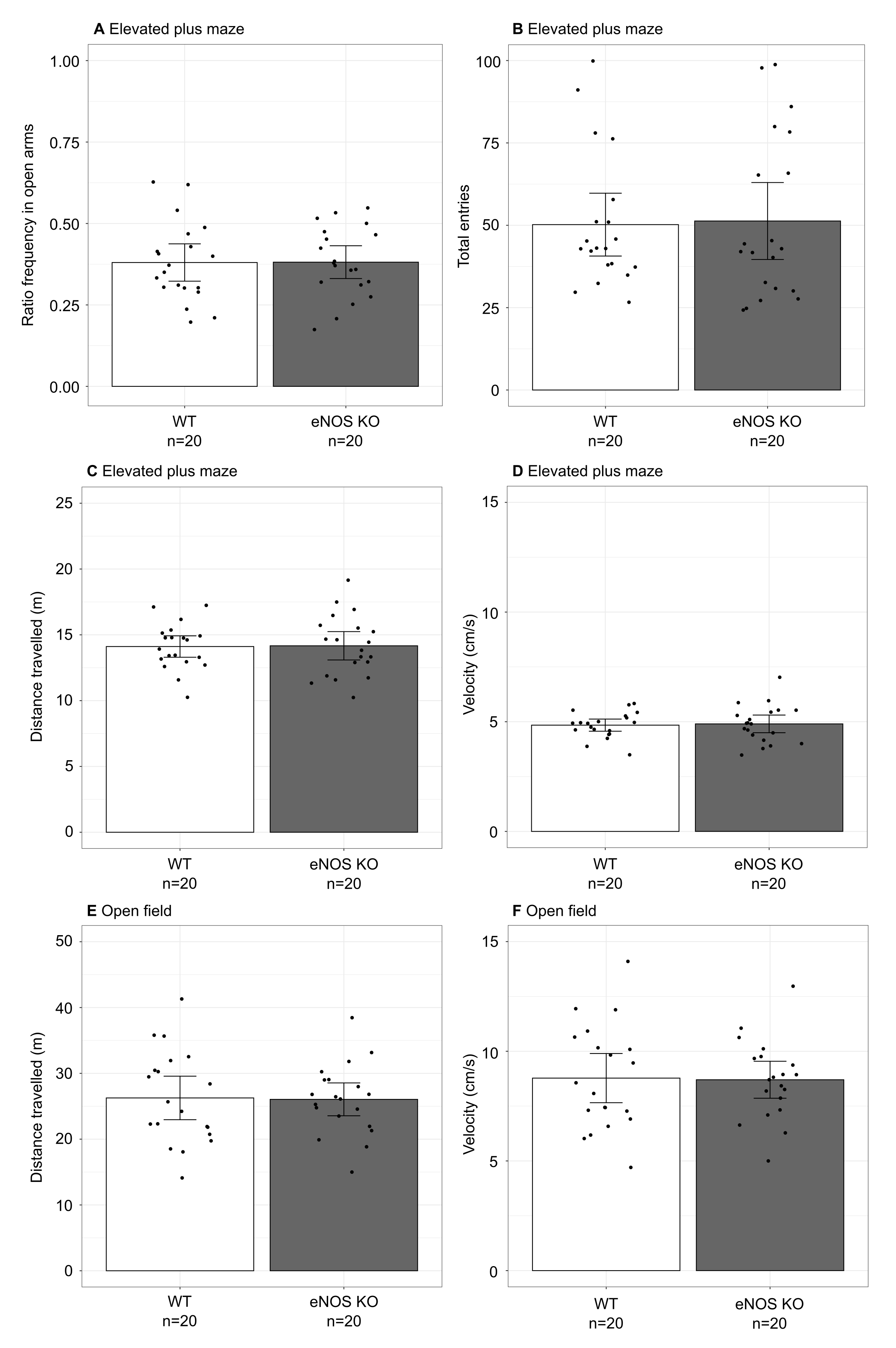

Supplement: Supplementary file 1 — Supplementary Figure 1. There were no significant differences between eNOS KO (gray bars) and WT mice (white bars) observed in the (a) ratio frequency of entries in the open arms of the elevated plus maze, (b) total entries (open or closed) in the elevated plus maze. There were also no significant differences between genotypes in the distance travelled in (c) the elevated plus maze or (e) the open field or in the mean velocity in (d) the elevated plus maze or (f) open field. Data shown as means and 95% confidence intervals. n refers to the number of mice (10 females and 10 males per group) [file BRB3-12-e2801-s001.png]
